# A New Sensitive Test Using Virtual Reality and Foam to Probe Postural Control in Vestibular Patients: The Unilateral Schwannoma Model

**DOI:** 10.3389/fneur.2022.891232

**Published:** 2022-05-25

**Authors:** Grâce Oussou, Christophe Magnani, Ioannis Bargiotas, Georges Lamas, Frederic Tankere, Catherine Vidal

**Affiliations:** ^1^Centre Borelli, CNRS UMR-9010, Université Paris Descartes, Paris, France; ^2^Department of ENT, Salpetriere Hospital, Paris, France

**Keywords:** video head impulse test, vertigo, hearing loss, visual moving scenes, vestibular nerve, vestibular-evoked myogenic potentials, EquiTest, calorics

## Abstract

Vestibular schwannomas (VS) are benign tumors of the vestibular nerve that may trigger hearing loss, tinnitus, rotatory vertigo, and dizziness in patients. Vestibular and auditory tests can determine the precise degree of impairment of the auditory nerve, and superior and inferior vestibular nerves. However, balance is often poorly quantified in patients with untreated vestibular schwannoma, for whom validated standardized assessments of balance are often lacking. Balance can be quantified with the EquiTest. However, this device was developed a long time ago and is expensive, specific, and not sensitive enough to detect early deficits because it assesses balance principally in the sagittal plane on a firm platform. In this study, we assessed postural performances in a well-defined group of VS patients. We used the Dizziness Handicap Inventory (DHI) and a customized device consisting of a smartphone, a mask delivering a fixed or moving visual scene, and foam rubber. Patients were tested in four successive sessions of 25 s each: eyes open (EO), eyes closed (EC), fixed visual scene (VR0), and visual moving scenes (VR1) delivered by the HTC VIVE mask. Postural oscillations were quantified with sensors from an android smartphone (Galaxy S9) fixed to the back. The results obtained were compared to those obtained with the EquiTest. Vestibulo-ocular deficits were also quantified with the caloric test and vHIT. The function of the utricle and saccule were assessed with ocular and cervical vestibular-evoked myogenic potentials (o-VEMPs and c-VEMPs), respectively. We found that falls and abnormal postural oscillations were frequently detected in the VS patients with the VR/Foam device. We detected no correlation between falls or abnormal postural movements and horizontal canal deficit or age. In conclusion, this new method provides a simpler, quicker, and cheaper method for quantifying balance. It will be very helpful for (1) determining balance deficits in VS patients; (2) optimizing the optimal therapy indications (active follow-up, surgery, or gamma therapy) and follow-up of VS patients before and after treatment; (3) developing new rehabilitation methods based on balance training in extreme conditions with disturbed visual and proprioceptive inputs.

## Introduction

Vestibular schwannomas (VS) are benign tumors that develop from the Schwann cells surrounding the vestibular nerve. They can cause nerve VIII dysfunction by blocking nerve vascularization or by compressing the auditory and vestibular nerve fibers (1). These benign, slow-growing brain tumors affect the quality of life of patients (2). Tumor growth can lead to hearing loss, tinnitus, and imbalance, and further size increases can lead to brainstem compression (3). The estimated annual incidence of these tumors ranges from 1 to 2 per 100,000 inhabitants, according to the French National Authority for Health (HAS). A review (4) revealed that most patients complained of dizziness before and after surgery. Evaluations of balance performance in patients with VS are an important part of clinical evaluations to determine the appropriate treatment such as surgery, gamma therapy or functional follow-up, and MRI (every 6 months or annually) according to tumor size and volume [stages I–IV from Koos classification (5)]. Assessments of the severity of balance impairment are potentially useful for identifying patients likely to benefit from vestibular rehabilitation and for adapting vestibular rehabilitation strategies to the needs of the patient (4). Several methods have already been developed for testing balance: the Romberg test (6), the EquiTest (7), and the Wii Balance Board (WBB) (8). Five years ago, we developed an application called BalanceRite, for quantifying WBB time series data on an iPhone or iPod Touch (9). We were also able to modify two sensory inputs involved in balance: visual inputs *via* virtual reality (VR) and proprioceptive inputs, by attaching foam rubber to the WBB. Using the WBB, we were able to measure postural stability during a visual or visual and proprioceptive (foam attached to the board) perturbation (9).

In this study, we developed a new method, not involving the WBB, for quantifying postural oscillations in patients subjected to visual and proprioceptive disturbances. This quantification was achieved with accelerometers from an Android smartphone (Galaxy S9) used as a sensor and placed on the lumbar vertebrae during the recordings on foam rubber. VS patients and controls were tested in four sets of conditions: eyes open (EO), eyes closed (EC), fixed visual scene (VR0), and moving visual scene (VR1) delivered *via* the mask. We also assessed the balance of the VS patients with the EquiTest. We characterized nerve VIII dysfunction, audiometric and vestibular function with caloric tests, vHIT, and cervical and ocular VEMPS (c- and o-VEMPs).

We had three aims: (1) To analyze the effects of VS on the vestibular system through caloric, vHIT, and VEMP assessments; (2) to study the effect of VS on balance performance with the DHI and our new device VR/Foam (foam + VR + smartphone); and (3) to demonstrate the advantages of VR/Foam over the EquiTest for assessing balance.

Soon, we hope to be able to determine postural performance for individual VS and try to develop new methods of vestibular rehabilitation. In addition, the vestibular tests combined with the VR/Foam should help the surgeon choose the best treatment (active follow-up of VS, Gamma Knife therapy, surgery). Indeed, GK may induce dizziness.

## Methods

In this study, we aimed to compare three categories of tests in the same patients to assess the functional deficit following a unilateral VS. They are summarized in Table 1. The first category of test encompassed the clinical signs (patients characteristics): age, body mass index (BMI), the VS stage according to Koss classification (5), and the dizziness Handicap Inventory (DHI). In essence, they are multimodal to the extent they mixed questionnaires, radiological signs, and morphologic variables. The second category of variables probed specifically the auditory and vestibular functions and therefore can be considered as unimodal tests. The audition was tested with standard tonal and vocal audiometry; the Horizontal semi-circular canal function was investigated with the caloric and vHIT tests, the otoliths function with c-VEMP and o-VEMP (see below and Table 1). Finally, the gold standard EquiTest and the newly designed VR/Foam test probed postural control in patients. Both tests are multimodal to the extent they probe how patients combined vestibular, visual, and proprioceptive information to overcome their vestibular deficit.

**Table 1:** The three categories of variables used to assess the functional deficit following a unilateral VS clinical signs (patients characteristics) gave multimodal informative data; auditory and vestibular unimodal tests allowed the appreciation of the dysfunction of either the auditory and or of the vestibular function; postural control which were by essence multimodal and which combined EquiTest and VR/Foam tests.

**Class of tests**	**Data**	**Uni or multimodal**
Patients characteristics	Age, BMI, VS Stage, DHI	Multimodal
Auditory and Vestibular tests	Hearing loss, Calorics, vHIT, c-and o-VEMPS	Unimodal
Postural control	EquiTest, VR/Foam	Multimodal

This retrospective study included a group of controls (*n* = 46) and a group of patients with unilateral vestibular schwannoma (*n* = 63). The protocol was approved by the local Ethics Committee following the requirements of the Helsinki Declaration. All subjects included in the study gave written and informed consent.

### The Healthy Controls

There were 30 female and 16 male controls, aged 16–90 years (mean age: 58 ± 17.9). None of the individuals complained of either dizziness or vertigo. Controls were only selected for this study if they had a dizziness score on the DHI < 20. The subjects were asked to provide their height and weight for the calculation of BMI. The healthy controls had normal results in vestibular and hearing tests (vHIT, audiogram). They were able to balance easily for 25 s on the VR/Foam in all four sets of test conditions and had normal scores in all EquiTest conditions, including conditions 5 and 6.

### Unilateral VS Group

The VS group consisted of 43 female and 20 male patients (*n* = 63). The VS was on the left side in 31 patients, and the right side in 32 patients. The patients were aged 20–86 years (mean age: 58.2 ± 15.5 years). DHI scores were >30 in 41.3% of the VS patients. All patients were diagnosed by brain MRI. Only patients with a brain MRI diagnosis of VS stage I, II, III, or IV according to the Koos classification were included in the VS group. BMI was calculated for each patient.

The DHI questionnaire developed by Jacobson and Newman (10) was used to assess the handicap suffered by the patients in terms of dizziness and unsteadiness. All subjects completed the DHI on a customized computer table, to provide a detailed evaluation of the degree of handicap due to balance or vertigo problems.

### Audiometric Tests

Tympanometry and stapedial reflex assessments were carefully performed, to exclude patients with conductive (even slight) hearing loss, to prevent the misinterpretation of air-conducted sound (ACS) o-VEMPs. The mean pure-tone threshold (PTA) for tones at 500 Hz, 1 kHz, and 2 kHz was used as an indicator of hearing loss.

### Vestibular Tests

#### vHIT: Horizontal and Vertical Canal Tests

The function of the horizontal semicircular canals at high frequencies of stimulations was assessed with horizontal vHIT from ICS Impulse (Otometrics/Natus, Denmark) as previously described for the head impulse paradigm (HIMP) (11). Subjects were instructed to focus on a fixed point on a wall at their eye level. The wall was 90 cm away. For each testing session, the clinician applied ~20 brief, rapid, horizontal head turns (head impulses) to each side, always starting from the center but of unpredictable timing and direction, with a minimal overshoot at the end of the head impulse. The amplitude of head rotation was ~10–15°, and the peak head velocity of the impulse was 180–220 deg/s, with angular accelerations of ~4,000–8,000 deg/s^2^ (9). Eye velocity and head velocity were recorded for each head turn. Gain is the usual measurement of the vestibulo-ocular reflex (VOR) slow phase in the HIMP paradigm: it represents the ratio of the eye and head areas under the curve during HIMP. Covert saccades were removed if they modified the gain of the horizontal vestibulo-ocular reflex (H-VOR). Gain values <0.8 are characteristic of a unilateral vestibular lesion (12).

#### Caloric Testing: Horizontal Canal Test

The function of the horizontal semicircular canals at low frequencies of stimulations was assessed with the caloric tests. They were performed with closed-loop sequential bithermal irrigation with water at 30 and 44 °C and video-nystagmography. The percent canal paresis (CP) was calculated with Jongkees' formula: CP = 100 ^*^ [(UW + UC) – (AW+AC)] / (UW + UC + AW + AC), where UW, UC, AW, and AC are the velocities of the induced ocular nystagmus obtained on the unaffected and affected sides, with warm and cold water, respectively. A value of CP >25% was considered to indicate an abnormal decrease on the affected side.

#### Otolith Function: Cervical and Ocular VEMPs

The function of the otolith system was assessed with the cervical and ocular VEMPs. Vestibular-evoked myogenic potentials were recorded with a Nicolet Viking 4 apparatus (Nicolet Biomedical Inc., Madison, WI, United States) with a four-channel averaging capacity, as previously described (13, 14).

*Cervical vestibular-evoked myogenic potentials* (cVEMPs) assess predominantly the function of the sacculo-spinal pathways (15). They were recorded from surface electrodes above the tensed sterno-cleido-mastoideus (SCM) muscle ipsilateral to the stimulated ear in response to air-conducted (AC) short-tone burst (STB) stimuli: 500 Hz, 102 dB nHL, 128 dB SPL, rise/fall time 2 m/s, plateau time 2 m/s, presented through calibrated TDH39 headphones. The EMG activity of the SCM was monitored on a display to ensure that sufficient muscle activation was maintained (>150 μV) (13). The latencies of the first two waves (P13 and N23) of the cVEMPs were measured in m/s, and the peak-to-peak amplitude between the P13 and N23 waves was measured in univolts (μV).

*Ocular vestibular-evoked myogenic potentials (oVEMP)* assess predominantly the function of the utriculo-ocular pathways (16). They were recorded from surface electrodes above the inferior oblique extraocular muscle contralateral to the stimulated ear in response to AC STBs. The AC STBs (500 Hz, 110 dB nHL, 132 dB SPL, rise/fall time 2 m/s, plateau time 2 m/s) were presented through calibrated TDH39 headphones. Patients with no measurable response on either side were considered to be non-responders. We measured the maximum latencies in m/s and the peak-to-peak amplitude in μV of the first two waves (n1 and p1) if the n1-p1 peak-to-peak amplitude was <2 μV.

The percent VEMP asymmetry in patients with unilateral vestibular lesions was measured by calculating the ratio of evoked potentials (EPr) as follows (17): EPr = 100^*^(Al – As)/(Al + As), where Al is the largest P13-N23 or n1-p1 peak-to-peak amplitude, and As is the smallest P13-N23 or n1-p1 peak-to-peak amplitude.

### Balance Test

The balance performance of the patients was tested in two ways: the newly designed VR/Foam test and the EquiTest. These two tests explore how patients compensate for a vestibular deficit by combining the remaining visual and proprioceptive information in various combinations.

#### Balance Quantification in the EquiTest

Balance was assessed with the sensory organization test (SOT) on the EquiTest (7, 1982). The SOT included six conditions delivered in a specific order, as follows. *Condition 1*: the subject was asked to stand upright whilst keeping his/her eyes open. *Condition 2*: the subject was asked to stand upright whilst keeping his/her eyes closed. *Condition 3***:** the cabin moved adaptively in response to the subject's movements. Here, the vision was sway-referenced. *Condition 4***:** the support base moved adaptively, following the subject's movements while the eyes were open: sway-referenced proprioception. *Condition 5*: as in condition 4, but with the eyes closed. *Condition 6*: the support base and the cabin moved in a synchronized manner: both vision and proprioception were sway-referenced. Based on the differences in body pressure center between the six different conditions, somatosensory, visual, and vestibular scores were calculated as percentages, a visual preference was estimated, and a composite score was obtained. Patients who did not fall but had a below-normal score were considered abnormal in conditions 5 and 6 of the EquiTest.

#### Balance Test on Foam With Moving Visual Scenes (VR/Foam)

**Figure 1:**
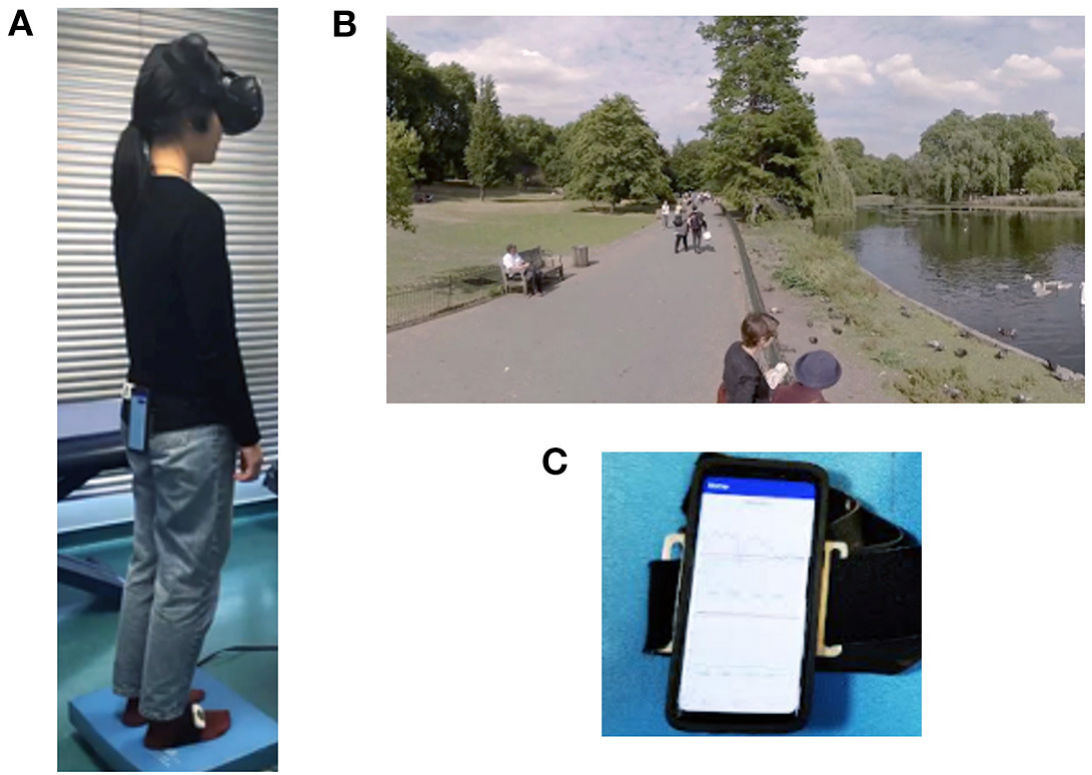
Illustration of the device for testing balance on foam rubber with VR. The subjects wear a mask over the eyes, which delivers a static or moving visual scene. They are asked to keep their balance for 25 s, and all postural oscillations along the *X, Y*, and *Z* axes are quantified with a smartphone attached to the subject's back. **(A)** Patient standing up on the foam with the smartphone one the back and the HTC Vive mask over the eyes. **(B)** Visual scan projected into the the mask. **(C)** Smartphone allowing the quantification of the postural oscillations.

The balance performance of the patients was tested on foam rubber using an HTC, VIVE VR masks using Unity 3D as the interface (18–20). We tested postural oscillations under the following conditions, presented sequentially and in this order: eyes open (EO), eyes closed (EC), within a stable environment (VR0), and, finally, with the VR mask delivering a moving visual scene with a small amplitude of disturbance (VR1) (Figure 1).

As in the EquiTest, the conditions were selected to make it increasingly difficult for the patients to keep their balance. The foam used was a blue Airex Balance Pad (Airex AG, Sins, Switzerland, 41 cm × 50 cm × 6 cm thick). Patients were placed feet 3 cm apart with their head in the center of two cameras delivering the moving visual scenes into the mask. In all test conditions, the test took 25 s to perform on the foam provided. If the patients fell in EC, VR0, or VR1 conditions, they were systematically retested in the same condition (i.e., two trials were systematically performed if the patient fell). Patients able to maintain their balance during this second trial were then allowed to move on to the next set of conditions. Patients who fell on the foam in VR1 conditions were then systematically retested in VR1 conditions, but on the ground, rather than on the foam (21). This made it possible to exclude postural phobias due to the use of visual inputs.

In this study, the VR environment was a visual scene of swans and ducks moving in a park in London (Source: “360/VR Master Series/Free Download/London Park Ducks Swans,” https://vimeo.com/215985064). The scene was recorded with a 360° camera. A virtual environment was created from this scene with the cross-platform game engine Unity (Unity Technologies). We gave this virtual environment the geometric appearance of a white sphere, into which we projected the London Park Ducks Swans scene, to obtain the final 360° virtual environment. The different rotation speeds used for visual disruption were programmed in the C# language with the cross-platform Visual Studio environment (Microsoft and Mono Project).

The *y*-axis rotation was the sum of three sine waves with frequencies of 0.4, 0.1, and 0.1 Hz and phase angles of 0°, 25°, and 0°, respectively. The *z*-axis rotation was the sum of three sine waves with frequencies of 0.5, 0.2, and 0.2 Hz and phase angles of 70°, 45°, and 90°. In each case, it was possible to control the peak amplitude of the rotation (between 0° and 30°), which was arbitrary, on a VR scale between 0 and 1. We chose VR1 condition because this visual moving scene triggered no falls in normal subjects on the foam other than elderly individuals over the age of 65 years (9).

**Figure 2:**
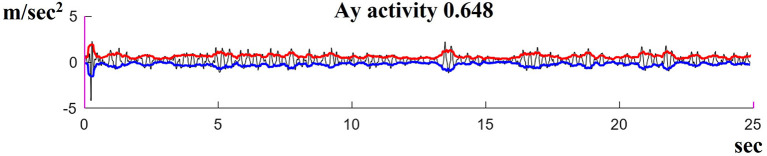
Illustration of the method used to quantify postural oscillations measured by the Android sensor along the *X, Y*, and *Z* axes in m/s^2^. A typical result in VR1 conditions is shown for a VS patient displaying high oscillations.

Postural oscillations were quantified with an Android smartphone (Galaxy S9) used as a sensor and held in position on the back by a belt at the level of the lumbar vertebrae. This device made it possible to measure the linear accelerations with the effect of gravity removed. Figure 2 shows an example of such a measurement. The black sinuous and noisy curve represents the acceleration along the *x, y*, and *z*-axis (in m/s^2^ = meters per second^2^) measured by the Android sensors (Galaxy S9) in the phone's reference frame. The red and blue curves were plotted in MATLAB as the RMS signal envelope of this acceleration. The area between the red and blue curves was used to estimate the amplitude of the oscillations. For the comparison of measurements, we defined the quantity “A*x*, A*y*, A*z* activity” as the area divided by the duration, which provided effective acceleration values for the study (in m/s^2^).

The acceleration values obtained with the sensors for the *y*-axis were higher and more directly related to postural oscillations than those for the *x*- and *z-*axes, in subjects trying to keep their balance on foam. The Shapiro–Wilk normality test showed our data to be normally distributed (non-significant *p*-values for all variables). We, therefore, used the mean acceleration values obtained for the *y*-axis with two times the standard deviation, in all four conditions, for the control group as the reference values (norms). These norms (means + 2SD) were used to define the upper limit of the normal range. The individuals in the control and VS groups were classified as normal if they were able to balance for 25 s (acceleration values strictly below the norm), abnormal (if they were able to balance for 25 s but had acceleration values greater than the norm), and fallers (if they fell within 25 s) on the foam.

### Statistical Analysis

Statistical analyses were performed with the statistical software RStudio version 3.6.1. For numerical data comparisons, we used the Chi-square test to analyze the significance of differences in balance assessments between the foam test and the EquiTest platform test. The Shapiro test showed that the data were normally distributed. Differences with *p* below 0.05 were considered to be significant. To identify the relationship between the tests and other parameters, correlation coefficients were calculated either with Pearson if the variables were raw numerical data or Spearman-Rho if the variables were not raw numerical data only.

## Results

As described in the method section, in this study, we aimed to compare the same patients with three categories of tests to assess their functional deficits following a unilateral VS. They are summarized in Table 1. The first category of tests encompassed the clinical signs or patients' characteristics, and the second category of variables probed specifically the vestibular functions. And the gold standard EquiTest and the newly designed VR/Foam test probed postural control in patients.

### The Clinical Signs

The patients' characteristics are the following: The VS patients were aged between 20 and 86 years (mean age: 58.2 ± 15.5). A total of 37 (58.7%) patients were younger than 65 years and 26 (41.3%) were older than 65 years (seniors). According to the Koos classification (5), 31 patients had VS stage I, 21 had VS stage II, 8 had VS stage III, and 3 had VS stage IV tumors. The mean BMI was 25.3 ± 4.12. The mean DHI score was 28.2 ± 24.2 and 26 of the 63 patients (41.3%) had a DHI score >30 (mean score for these patients: 51.3 ± 14.2, with a minimum score of 30 and a maximum score of 78).

### The Auditory and Vestibular Function

#### Auditory Test

A total of 50 out of 63 (79.4%) patients had an abnormal hearing function on the VS side. The mean hearing loss was 63.24 dB ± 24.1.

#### Vestibular Function

Vestibular dysfunction of the horizontal canal nerve was assessed by both caloric tests and the vHIT (H).

##### Caloric Tests

The results of the caloric test were abnormal on the side of the lesion in 42 of the 63 patients (66.6%), with a mean deficit on the VS side of 64.7% ± 24.6 (canal paresis range: 29–100%).

##### Horizontal vHIT

The results of the vHIT were abnormal on the side of the lesion in 19 of the 63 patients (30.2%), with a mean gain deficit for horizontal canal function of 0.61 (range 0.3–0.77) with covert and overt saccades.

**Figure 3:**
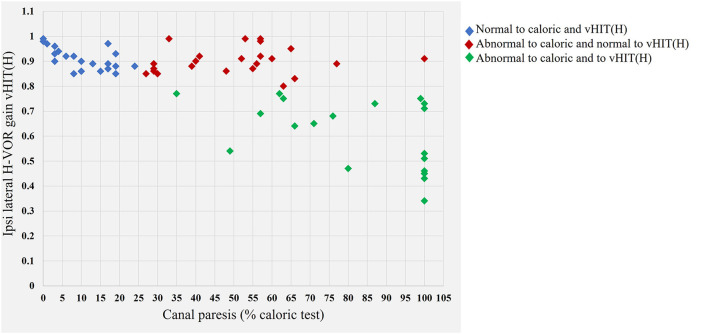
Graph illustrating the relationship between the horizontal vestibulo-ocular gain in the vHIT test and canal paresis in the caloric test. Blue diamonds indicate the normal values for the caloric test and vHIT (H). Red diamonds indicate values for patients with a normal vHIT result but abnormal caloric test results (canal paresis >25%). A dissociation is observed between the findings for the two different tests of horizontal nerve function [normal vHIT (H)—abnormal canal paresis]. Green diamonds, abnormal results in both the caloric test and vHIT (H).

No correlation was detected between mean vHIT (H) gain on the injured side and the caloric test result (Figure 3). More importantly, 23 of the 63 (36.5%) patients had a normal vHIT (H) result (gain > 0.85) but abnormal caloric test results (see Table 2 and Figure 3 red diamonds). The patients with this dissociation had a mean caloric deficit of 50.6% ± 17.9. We identified no patients with abnormal vHIT (H) and normal canal paresis results.

**Table 2:** Data for the 23 of VS 63 patients presenting a dissociation between the average VOR gains of the three canals tested with the vHIT (H) and the results of the caloric test.

**VS patients**	**Side**	**vHIT (H)**	**vHIT (P)**	**vHIT (A)**	**Canal paresis %**
Patient 1	L	0.85	0.85	0.92	27
Patient 2	R	0.9	0.9	0.8	29
Patient 3	R	0.89	0.88	0.89	29
Patient 4	L	0.86	0.98	0.98	29
Patient 5	R	0.86	0.9	0.86	30
Patient 6	R	0.99	0.95	0.72	33
Patient 7	R	0.86	0.85	0.89	39
Patient 8	L	0.9	0.76	0.88	40
Patient 9	R	0.87	0.89	0.96	41
Patient 10	L	0.86	0.76	0.86	48
Patient 11	R	0.89	0.9	0.96	52
Patient 12	R	0.99	0.62	0.73	53
Patient 13	L	0.87	0.96	0.83	55
Patient 14	R	0.90	0.99	0.89	56
Patient 15	R	0.99	0.89	0.89	57
Patient 16	R	0.88	0.57	0.88	57
Patient 17	R	0.99	0.57	0.98	57
Patient 18	R	0.91	0.4	0.81	60
Patient 19	L	0.8	0.5	0.9	63
Patient 20	L	0.95	0.91	0.89	65
Patient 21	L	0.83	0.9	0.89	66
Patient 22	R	0.89	0.92	0.9	77
Patient 23	R	0.91	0.84	0.74	100

*Seven of the 23 patients had a deficit for the mean gain of the posterior ROV (Patients 8, 10, 12, 16, 17, 18, 19) and 3/63 presented isolated decreases in anterior ROV gain (Patients 6, 12, 23). All 10 patients with abnormal caloric tests had normal vHIT (H) results. Side, the side on which the VS was located, left (L) or right (R); vHIT (H), mean gain of the H-ROV; vHIT (P), posterior gain; vHIT (A), anterior gain; canal paresis %, quantification of the deficit in the caloric test*.

**Figure 4:**
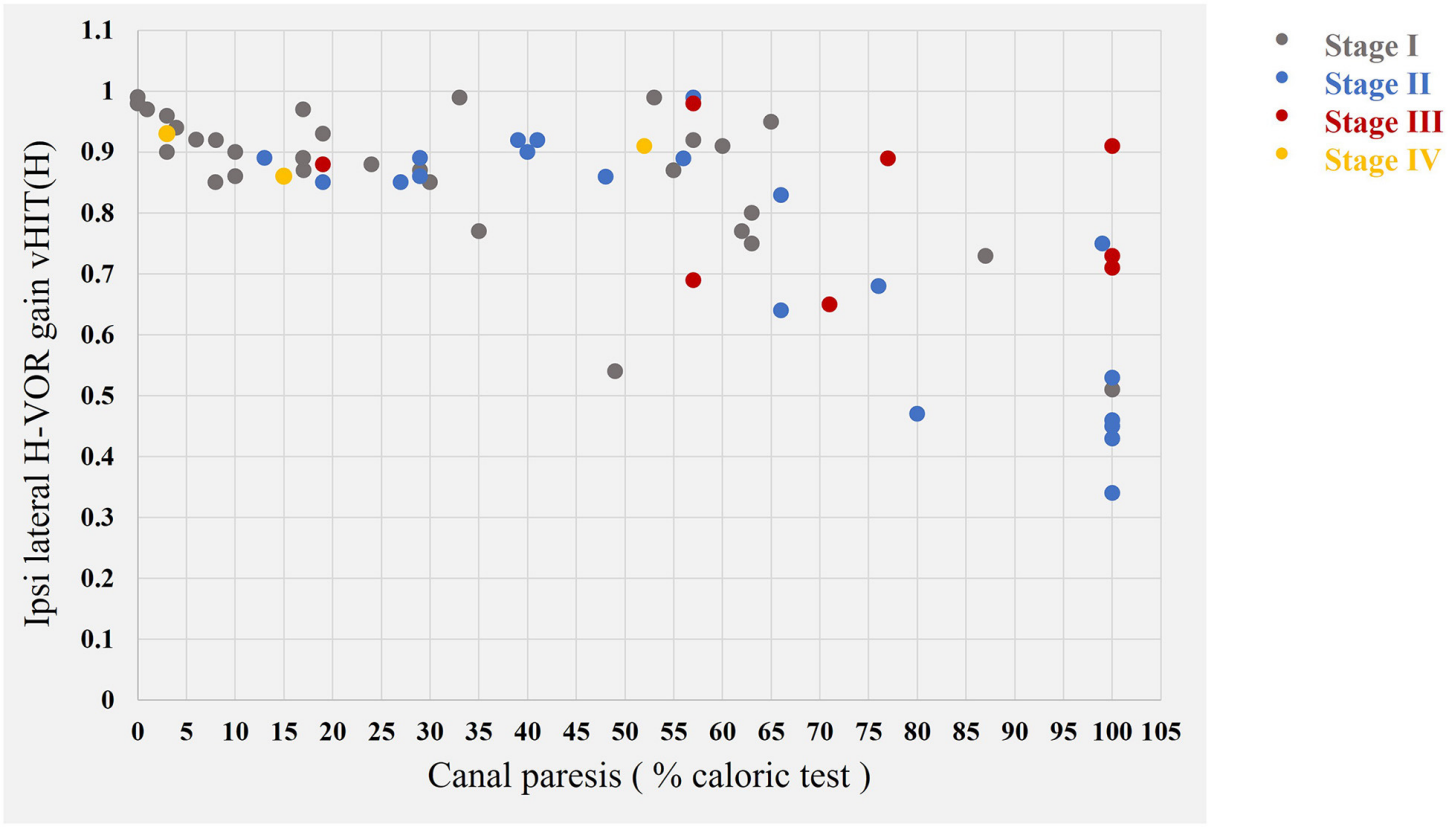
Representation of the results obtained with the vHIT (H) and caloric tests as a function of VS stage. In gray, the 31 stage I tumors; in blue, the 21 stage II tumors; in red, the 8 stage III tumors, and, in yellow, the 3 stage IV tumors according to the Koos classification (5).

We found no correlation between VS stage and vHIT (H) (correlation coefficient: - 0.159) and also VS stage and caloric test results (correlation coefficient: 0.386) (Figure 4).

##### Anterior and Posterior vHIT

Isolated posterior canal vHIT (vHIT P) was abnormal in 24/63 patients (38.1%) and isolated anterior canal vHIT (vHIT A) was abnormal in 16/63 patients (25.4%). Abnormal VOR gain for all anterior, posterior, and horizontal canals was detected in 12 of 63 patients (19%). All 12 patients also had abnormal canal paresis on caloric testing (mean canal paresis 85.3 ± 20.9%).

**Figure 5:**
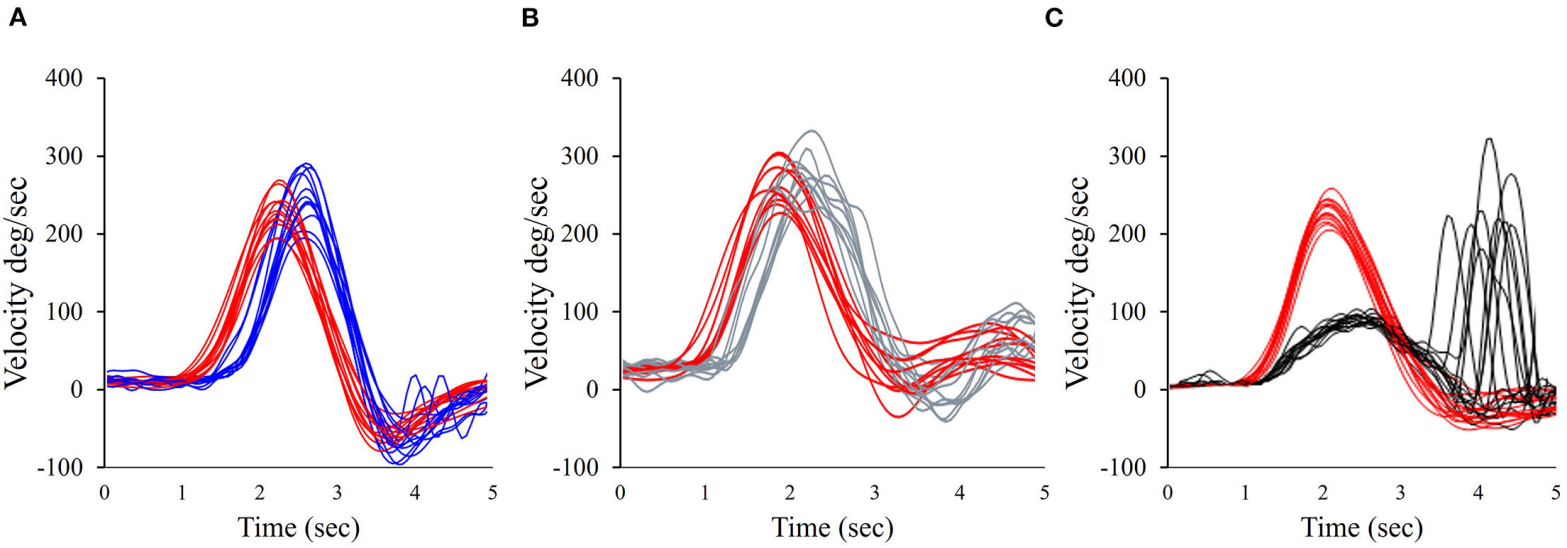
Illustration of the vHIT for patient 18 in Table 2. Note the normal horizontal VOR gain; eye velocity is shown in blue, and head velocity is in red; the normal vHIT (A) eye velocity is shown in gray, with head velocity in red. This patient has an abnormal vHIT (P) for eye velocity in black and head velocity in red. **(A)** Horizontal vHIT; **(B)** Anterior vHIT; **(C)** Posterior vHIT.

Three of the 23 patients with dissociated vHIT (H) (normal) and caloric (abnormal) test results (13%) had an abnormal anterior gain and seven of the 23 (30.4%) had an abnormal posterior canal gain (Table 2). For example, Figure 5 illustrates the vHIT recording for patient 18 (from Table 2). Note the normal horizontal and anterior, but abnormal posterior VOR gain (Figure 5).

##### Otolith Saccular and Utricular Nerve Function

Hearing function was measured before VEMPs: hearing function was abnormal on the side of the VS in 50 of the 63 (79.4%) patients. Mean hearing loss was 63.2 dB ± 24.1. None of the patients had a conductive hearing loss.

The cVEMPs induced by STBs were abnormal on the side of the VS lesion (abolished or decreased) in 38 of the 63 patients (63.3%). The oVEMPs were also abnormal (abolished or decreased) on the side of the VS lesion in 29 of 63 patients (46%). Ocular VEMPs were normal in 24 patients (38.1%) and bilaterally suppressed in 10 patients (15.9%).

Among the 63 VS patients, 76% had abnormal c- and o-VEMPS, 66% abnormal caloric, and 47% abnormal vHIT. No significant difference could be found between caloric and VEMPs (*p* = 0.426).

### Postural Control

#### The EquiTest

All the healthy controls had normal balance in all the conditions of the EquiTest, including, in particular, conditions 5 and 6 (*n* = 46).

In the VS group, in condition 5 of the EquiTest, 18 of the 63 patients fell (28.6%), and 20 patients (31.7%) had an abnormal score but did not fall. The remaining patients had normal performances in the three trials. In condition 6 of the EquiTest, 27 of the 63 patients (42.9%) fell and 2/63 patients (3.2%) had an abnormal score but did not fall. In summary, 38 of the 63 VS patients (60.3%) fell or had an abnormal score in condition 5, and 29/63 patients (46%) fell or had an abnormal score in condition 6. The abnormal scores and frequencies of falls in EquiTest conditions 5 and 6 were highly significant, with a *p* = 0.0009. Eighteen of 63 patients (28.6%) fell in both conditions 5 and 6. All VS patients who fell in condition 5 of the EquiTest also fell in condition 6.

#### EO, EC, and VR Tests on Foam

For the healthy group, the mean values for AY activity in m/s^2^ obtained on the foam for EO, EC VR0, and VRI are presented in Table 3. The mean ± SD was 0.10 ± 0.03 for EO, 0.18 ± 0.03 for EC, 0.12 ± 0.04 for VR0, and in 0.28 ± 0.06 for VR1.

**Table 3:** Presentation of the mean values (means + standard deviations) obtained for each of the *X, Y*, and *Z* axes in the control group, in EO, EC, VR0, and VR1 conditions, on foam, with smartphone sensors attached to the back.

**Conditions**	**Activity (m/s^**2**^)**	**Mean**	**SD**
**Foam** **+** **EO**	AX activity	0.080	0.034
	AY activity	0.103	0.034
	AZ activity	0.080	0.036
**Foam** **+** **EC**	AX activity	0.129	0.052
	AY activity	0.187	0.034
	AZ activity	0.134	0.054
**Foam** **+** **VR0**	AX activity	0.089	0.039
	AY activity	0.123	0.041
	AZ activity	0.086	0.034
**Foam** **+** **VR1**	AX activity	0.204	0.074
	AY activity	0.282	0.060
	AZ activity	0.200	0.090

The AY activity norms (mean + 2SD) were 0.17 for EO, 0.25 for EC, 0.20 for VR0, and 0.40 for VR1. No falls were detected in the healthy group, even in the two most difficult conditions: EC and VR1.

**Figure 6:**
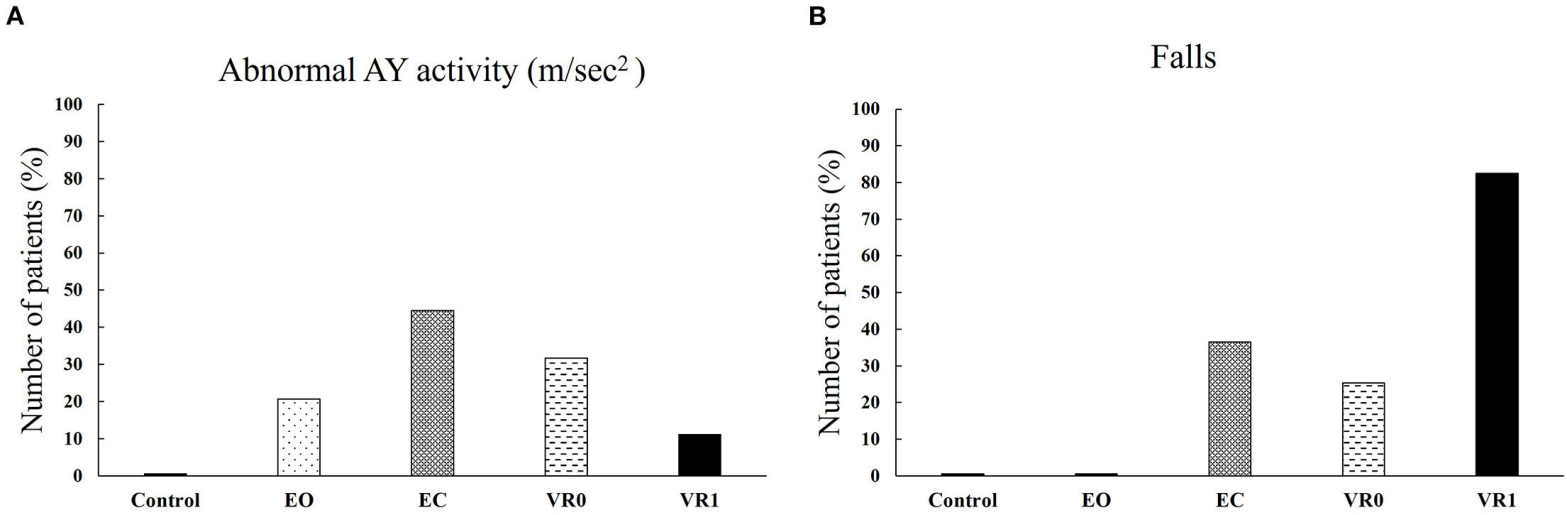
Percentage of patients displaying abnormal postural oscillations or falling, for the control and VS groups, under EO, EC, VR0, and VR1 conditions. **(A)** Percentage of patients with abnormal AY activity in control group an VS group in the EO, EC, VR1 and VR1 conditions; **(B)** Percentage of falling in control in control group an VS group in the EO, EC, VR0 and VR0 conditions.

**Figure 7:**
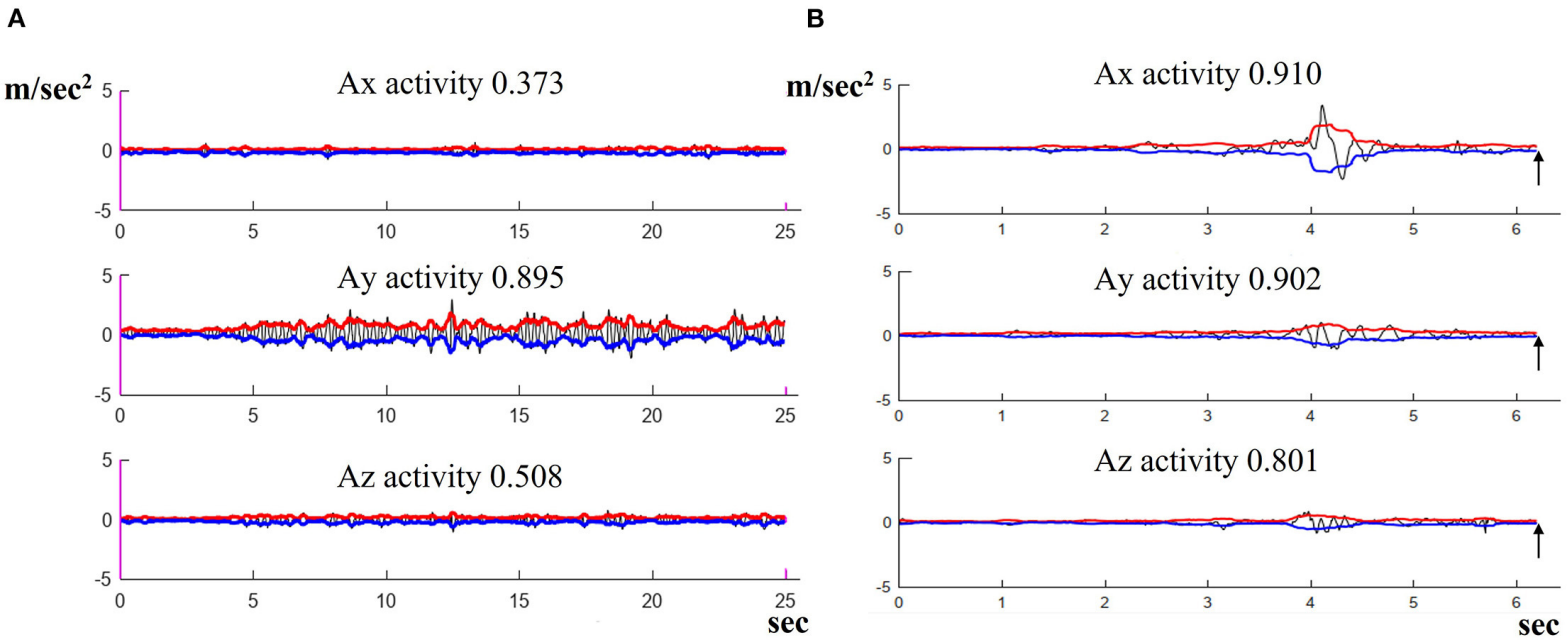
Illustration of the oscillations of acceleration (m/s^2^) along the *X, Y*, and *Z* axes, as measured with the Android sensor. **(A)** Abnormal result in VR1, not the high AY activity in m/s^2^, with large oscillations. **(B)** A fall, about 6 s after the start of the recording (see arrow).

Postural oscillations were larger than normal in 13 of the 63 patients (20.6 %) for EO and 28 of the 63 patients (44.4%) for EC (*p* = 0.00315). Similarly, large oscillations were observed in 20/63 patients (31.7%) for VR0 and 7/63 patients (11%) for VR1 (*p* = 0.0015) (Figures 6A, 7A).

Falls were observed in 0/63 VS patients (0%) in EO conditions and 23/63 patients (36.5%) in EC conditions which is statistically different (*p* = 1.527e−09). Falls were significantly more frequent in VR1 conditions 52/63 patients 82.5%, (*p* = 3.863e−08) than in VR0 conditions (16/63 patients, 25.4%) (Figure 6B). VR1 was the most destabilizing set of conditions. Figure 7B shows the recording of patients who fell after 6.2 s.

We found no correlation between age and the likelihood of falling (correlation coefficient: −0.035). Some patients over 65 years of age did not fall, whereas other much younger patients fell within 10 s. The time to the fall ranged from 3 to 20 s for EC, 1 to 21 s for VR0, and 1 to 17 s for VR1. A 75% of the VS patients who fell did so within 10 s in VR1 condition. Some VS patients displayed large oscillations and had abnormal AY activity values during the VR/Foam test but did not fall (Figure 6B).

No falls or abnormal postural oscillations were detected in patients who performed the VR1 test on the ground (a systematic VR1 test on the ground was performed for all those who fell two times during VR1 tests on foam; see Methods).

We also found no correlation between abnormal caloric test results and falls in VR/Foam conditions. There was also no correlation between cVEMPs and oVEMPs with falls in VR/Foam conditions (the correlation coefficient: 0.28). More importantly, no correlations between all these multi- and unimodal tests and the result of the VR/Foam test could be found.

#### EquiTest vs. VR/Foam

**Figure 8:**
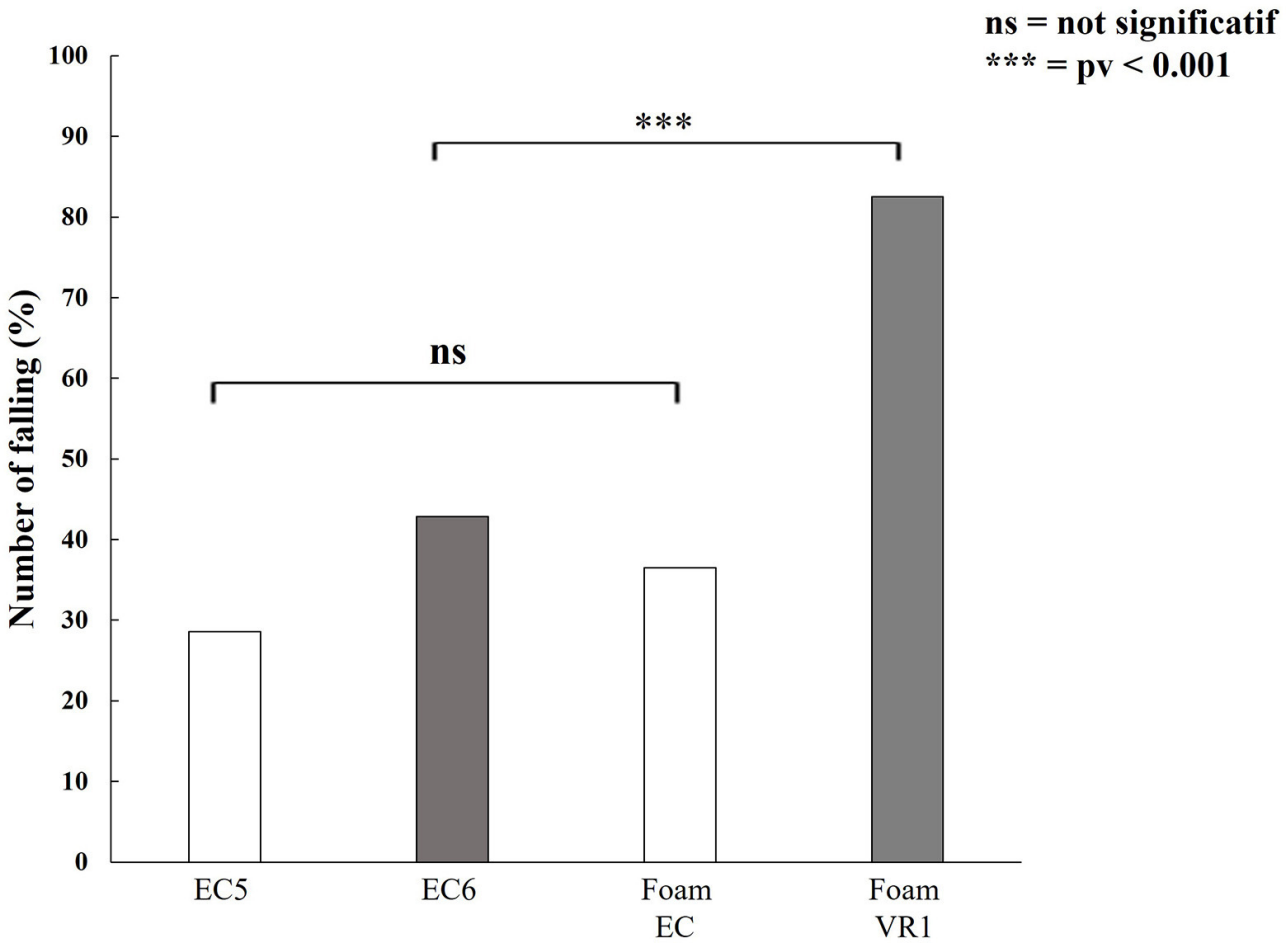
Percentage of patients falling in the EquiTest (EC5, condition 5 and EC6, condition 6) and with our VR-foam-smartphone device. Note that VR1 is much more sensitive than EquiTest for detecting an imbalance in patients suffering from a unilateral VS.

We found no significant difference between the percentages of patients falling in conditions 5 and 6 of the EquiTest (*p* = 0.2). By contrast, there was a highly significant difference (*p* = 0.000024) between the percentages of patients falling in EC and VR1. A highly significant difference was found between EquiTest condition 6 and the VR1 condition of balance on foam: 82.5% of the VS patients fell in VR1 conditions, whereas only 42.9% fell in condition 6 of the EquiTest (*p* = 0.0003) (Figure 8). No significant difference in the percentage of patients falling or in abnormal values was detected between EC on foam and condition 5 of the EquiTest (*p* = 0.9).

## Discussion

We show in this study, for the first time, that patients with unilateral VS frequently present balance abnormalities when placed in highly disturbed conditions (distortion of visual and proprioceptive information), regardless of their age, vestibular system function, or vestibular compensation processes.

### DHI and Balance Tests

Many VS patients complain of instability and poor balance in their daily lives (4). These complaints were revealed in the results of the DHI questionnaire (22), with 41.3% of VS patients having a DHI score >30. We found no correlation between the patients' complaints and the results of the various balance tests performed. There was also no relationship between the results of the vestibular tests and the DHI score. This lack of correlation probably reflects an inability of the patients to self-assess their balance state accurately. The DHI questionnaire combines functional, physical, and emotional questions. However, it does not measure the psychological state and anxiety symptoms of the patients (fear of falling), which may also have an effect on the appreciation of balance by the patient.

### VS and Vestibular Function

Caloric test results were abnormal on the side of the lesion in 67% of the VS patients. The superior vestibular nerve was, therefore, frequently affected by the schwannoma, regardless of tumor stage. In our patients, horizontal canal paresis, when present, was well compensated, as no ocular nystagmus was detected when the patients opened their eyes in darkness while in the supine position. The growth of this benign tumor was very slow [ <2.5 mm per year (23)], accounting for the effective compensation of static ocular deficits when the tumor was detected by MRI, many years after disease onset in some cases.

By contrast, abnormal vHIT (H) results were obtained for only 30.2% of the patients, consistent with a previous report (24). Caloric irrigation was shown to have a higher sensitivity than vHIT [72% *vs*. 41% (25)]. We obtained a sensitivity of 66.7% for caloric irrigation and 30.2% for vHIT. This dissociation may reflect the intrinsic differences between these two tests: the caloric test evaluates the horizontal vestibular system in the low-frequency range (0.003 Hz) (26), whereas vHIT (H) assesses the function, not only of the horizontal vestibular system, but also of the anterior and posterior vestibular system (through head impulses) up to 5 Hz (27). Furthermore, different mechanisms are at work in the stimulation of the vestibular hair cells of the horizontal ampullae in these two tests: in the caloric test, the cells are stimulated by endolymph flow due to a temperature gradient from one side of the canal to the other (28). The head impulse induces a flow of endolymph due to the high acceleration, low amplitude, and high velocity of head impulses on either side (29). The explanation for this discrepancy remains unclear: it could be due to the caloric test preferentially evaluating tonic fibers over phasic fibers. Some neurons (tonic neurons) have been shown to display a sustained response to maintained stimuli, whereas others (phasic neurons) have a transient response to the same stimuli. A similar distinction has been applied to the primary semicircular canal (30), which establishes diverse vestibular afferences (31). Tonic vestibular primary neurons may be more sensitive to the temperature gradient in the caloric test. The vHIT should be used in addition to the caloric test, as it can be used for anterior and posterior evaluations in addition to quantifying horizontal canal nerve function.

The vHIT is a useful bedside examination of the semicircular canals, because it provides information about sensitivity to higher frequencies of the vestibular–ocular reflex (VOR) <0.002 Hz for caloric testing and up to 0.8 Hz for vHIT (32). Neurons synapsing with the type I receptors of canals have also been reported to display a preference for high frequencies, which has been interpreted as sensitivity to shaking (33).

It has also been suggested that some patients with VS also suffer from dysfunctions of the secretion and resorption of endolymphatic liquid, potentially with associated hydrops (34) due to the inflammatory process occurring around the vestibular schwannoma (35). None of our subjects displayed an abnormal vHIT result with a normal caloric test result. Similar findings have been reported for other vestibular conditions, such as vestibular neuritis and chemical labyrinthectomy. We found no correlation between VS stage and the degree of canal paresis in caloric tests. The dissociation of the results of these two tests was not, therefore, related to tumor size or volume.

These two tests [caloric and vHIT (H)] can, therefore, be considered complementary. Furthermore, vHIT results could be normal for head impulses performed in the horizontal plane but abnormal for head impulses in the sagittal plane. For example, we found posterior (30.4%) and anterior (13%) canal dysfunction (albeit less frequently) in patients with a normal horizontal vestibulo-ocular reflex gain in the vHIT (H) test and abnormal results for the caloric test.

### Vestibular-Evoked Myogenic Potentials

The c- and o-evoked potentials were, therefore, also used to assess the probable vestibular deficit in these patients (13) that was not picked up by the caloric test. It is therefore essential to integrate all these vestibular otolith tests, to identify the vestibular deficits of the saccular and utricular nerves.

### Assessment of the Balance of VS Patients on the VR/Foam Device

We tested the VS patients with VR/Foam and the sensors of an Android smartphone (see Methods). We aimed to improve the detection of their deficits and to obtain a precise evaluation of their balance performances before making decisions about management (active follow-up, surgery, or gamma treatment).

Virtual reality proved to be a very good test for assessing the ability of the patient to maintain balance on foam under difficult and extreme conditions in which erroneous visual and proprioceptive information was supplied. This has already been shown to be the case for normal, senior, and bilateral areflexic patients (36). Furthermore, previous studies have also shown that unilateral vestibular dysfunction can lead to high sensitivity to visual stimulation (37) and a disturbance of proprioceptive inputs to the lower limbs (21).

In the VS group, the percentage of patients falling increased with the difficulty of the conditions, from EO to VR1: no falls were observed in EO but 36.6% of the VS patients fell in EC on the foam. Falls with the eyes closed could not be attributed to age in this study as no falls in EC conditions were detected in our control group, which included senior patients. Chiarovano et al. showed that <3% of healthy subjects over the age of 70 years fall into EC conditions (9). VS patients found it more difficult to keep their balance when they received disturbed proprioceptive inputs (due to standing on foam). Only two of our 63 VS patients were in their eighties.

Falls were observed in 25.39% of the VS patients in VR0 conditions and 82.53% in VR1 conditions. Age did not affect the likelihood of falling in VR1. Chiarovano et al. showed that falls occurred in VR1 conditions for 26% of healthy subjects over the age of 70 years and 66% of those over the age of 80 years (9).

This high percentage of falls in VR1 highlights the attentional demands on the patient for the maintenance of balance for 25 s on foam. Foam is used clinically as a tool for assessing the contribution of proprioceptive information to static postural control (38). VS patients were unable to use their vestibular system properly to ignore visual inputs, as standing on foam provided them with false proprioceptive information. They tried to keep their balance, but they often fell within 10 s (see Results). In 1982; Nashner et al. suggested that the main problem of patients with vestibular deficits was their inability to integrate sensory information (39, 40). Our patients appear to be less handicapped by the loss of vestibular information than by their inappropriate responses to proprioceptive and visual information (41). In our study, even patients with normal caloric test and VEMP results fell or had abnormal results in VR1. All our VS patients with normal caloric test results (<25%) fell or had abnormal results in VR1. All these patients had a history of rotatory or positional vertigo. They had, therefore, developed a dependence on visual cues, which was evident from the abnormal results and falls observed in VR1. It has been shown that following sensory loss, for example, unilateral vestibular deafferentation, individual weighting changes with the prevailing view that visual inputs become more important which often leads to high visual dependence (42).

In patients with abnormal results for VR1, AY activity levels were higher than those for AX and AZ activity, but with these large oscillations maintained until the end of the test without falling. Our patients who fell two times in VR1 conditions on foam had no problem when they were tested in VR1 on the ground because this made it possible for them to use true proprioceptive inputs correctly. In the presence of disturbances of proprioceptive and vision inputs, these patients required symmetric vestibular inputs to maintain their balance on the foam.

### VR/Foam vs. EquiTest

Condition 6 of the EquiTest is performed on firm ground support that swings exclusively in the anteroposterior plane (43). The patient's eyes are also open and receive sway-referenced vision input from the surrounding cabin. By contrast, VR1 on foam is performed with a total immersion in a virtual environment that very closely resembles reality (44). Furthermore, proprioception on foam is controlled in all the planes (anterior–posterior, inferior–superior, and lateral).

In the EquiTest, only 42.8% of patients fell in condition 6 due to their reliance on visual cues (45). VR1 conditions had a sensitivity of 93%, whereas EquiTest condition 6 had a sensitivity of 46%. Both tests had a specificity of 100% for both our groups (control and VS patients). These findings highlight the utility of our VR1 conditions (44). Abnormal results in the VR/Foam test are of particular interest because they are predictive of a high probability of falling. Such tests would therefore be useful for assessment before treatment or rehabilitation to improve the patient's balance.

### VR/Foam vs. the Other Unimodal Tests

No correlations between all the vestibular tests and the result of the VR/Foam test could be found. These results can be explained in two ways:

The VR/Foam probes how patients used a combination of visual proprioceptive and vestibular information, which may explain why we did not find any correlation in any specific vestibular tests we used. The underlying assumption would be that various degrees of vestibular compensation for each patient blurred a correlation between the VR/Foam test (multimodal tests and any specific vestibular test (unimodal test).

On the other hand, this argument cannot be put forward for the EquiTest, which is also a multimodal test like the VR/Foam. The present data cannot explain why VR/Foam is more sensitive than the EquiTest. Two hypotheses can be put forward but they should be tested in future studies:

First, standing on a foam may be more difficult than standing on a firm platform which always moves along the same axis of rotation.

Second, stabilizing the visual scene with respect to the head (EquiTest) could be less disturbing than a random movement of the visual scene with respect to the head (VR/Foam).

Finally, the combination of the visual scenes combined with the random disturbances caused by the foam support could be more disturbing than the more stereotyped sensory postural and visual conflict of the EquiTest. This hypothesis should be tested in further studies.

## Conclusions

Our findings demonstrate that VS affects the function of the superior and inferior vestibular nerve. Both caloric tests and vHIT should be performed to characterize the function of the horizontal canal nerve in patients suffering from VS. Finally, the test developed here, based on the use of foam, a VR mask, and smartphone sensors, is a highly sensitive and cheap method for quantifying balance. This test can detect postural performance disorders and visual dependence early, which is not always the case for EquiTest. This VR/Foam test should, therefore, help clinicians to assess and quantify balance performance. This system can also detect inappropriate vestibular compensation processes, such as visual dependence.

## Data Availability Statement

The raw data supporting the conclusions of this article will be made available by the authors, without undue reservation.

## Ethics Statement

Ethical review and approval was not required for the study on human participants in accordance with the local legislation and institutional requirements. The patients/participants provided their written informed consent to participate in this study.

## Author Contributions

CV directed the research. GO and CV conceived and performed all the balance tests for patients and wrote the paper. CM performed the mathlab work for X,Y,Z postural oscillation analysis. IB helps for sway velocity calculation. GL and FT helped to test the patients suffering from unilateral vestibular schwannoma. All authors have reviewed the text and approved the final paper for submission.

## Conflict of Interest

The authors declare that the research was conducted in the absence of any commercial or financial relationships that could be construed as a potential conflict of interest.

## Publisher's Note

All claims expressed in this article are solely those of the authors and do not necessarily represent those of their affiliated organizations, or those of the publisher, the editors and the reviewers. Any product that may be evaluated in this article, or claim that may be made by its manufacturer, is not guaranteed or endorsed by the publisher.

## References

[1] Wiegand DA Ojemann RG Fickel V. Surgical Treatment of Acoustic Neuroma (Vestibular Schwannoma) in the United States. Laryngoscope. (1996) 106:58–66. 10.1097/00005537-199601000-00012 8544629

[2] Propp JM McCarthy BJ Davis FG Preston-Martin S. Descriptive epidemiology of vestibular schwannomas. Neuro-oncol. (2006) 8:1–11. 10.1215/S1522851704001097 16443943 PMC1871924

[3] Saman Y Bamiou D-E Gleeson M. A contemporary review of balance dysfunction following vestibular schwannoma surgery. Laryngoscope. (2009) 119:2085–93. 10.1002/lary.20648 19806649

[4] Saman Y Bamiou D-E Murdin L Tsioulos K Davies R Dutia MB. Balance, Falls Risk, and Related Disability in Untreated Vestibular Schwannoma Patients. J Neurol Surg B Skull Base. (2014) 75:332–8. 10.1055/s-0034-1372469 25276598 PMC4176536

[5] Koos WT Day JD Matula C Levy DI. Neurotopographic considerations in the microsurgical treatment of small acoustic neurinomas. J Neurosurg. (1998) 88:506–12. 10.3171/jns.1998.88.3.0506 9488305

[6] Gschwind YJ Kressig RW Lacroix A Muehlbauer T Pfenninger B Granacher U. A best practice fall prevention exercise program to improve balance, strength/power, and psychosocial health in older adults: study protocol for a randomized controlled trial. BMC Geriatr. (2013) 13:105. 10.1186/1471-2318-13-105 24106864 PMC3852637

[7] Nashner LM. A model describing vestibular detection of body sway motion. Acta Otolaryngol. (1971) 72:429–36. 10.3109/00016487109122504 5316344

[8] Clark RA Bryant AL Pua Y McCrory P Bennell K Hunt M. Validity and reliability of the Nintendo Wii Balance Board for assessment of standing balance. Gait and Posture. (2010) 31:307–10. 10.1016/j.gaitpost.2009.11.012 20005112

[9] Chiarovano E Wang W Rogers SJ MacDougall HG Curthoys IS de Waele C. Balance in Virtual Reality: Effect of Age and Bilateral Vestibular Loss. Front Neurol. (2017) 8:5. 10.3389/fneur.2017.00005 28163693 PMC5247457

[10] Jacobson GP Newman CW. The development of the Dizziness Handicap Inventory. Arch Otolaryngol Head Neck Surg. (1990) 116:424–7. 10.1001/archotol.1990.01870040046011 2317323

[11] MacDougall HG McGarvie LA Halmagyi GM Rogers SJ Manzari L Burgess AM. A new saccadic indicator of peripheral vestibular function based on the video head impulse test. Neurology. (2016) 87:410–8. 10.1212/WNL.0000000000002827 27251884 PMC4977115

[12] MacDougall HG McGarvie LA Halmagyi GM Curthoys IS Weber KP. The Video Head Impulse Test (vHIT) detects vertical semicircular canal dysfunction. PLoS ONE. (2013) 8:e61488. 10.1371/journal.pone.0061488 23630593 PMC3632590

[13] Chiarovano E Darlington C Vidal P-P Lamas G Waele C de. The role of cervical and ocular vestibular evoked myogenic potentials in the assessment of patients with vestibular schwannomas. PLoS ONE. (2014) 9:e105026. 10.1371/journal.pone.0105026 25137289 PMC4138161

[14] Chiarovano E Vidal P-P Magnani C Lamas G Curthoys IS de Waele C. Absence of rotation perception during warm water caloric irrigation in some seniors with postural instability. Front Neurol. (2016) 7:4. 10.3389/fneur.2016.00004 26834699 PMC4725157

[15] Colebatch JG Halmagyi GM Skuse NF. Myogenic potentials generated by a click-evoked vestibulocollic reflex. J Neurol Neurosurg Psychiatry. (1994) 57:190–7. 10.1136/jnnp.57.2.190 8126503 PMC1072448

[16] Curthoys IS Iwasaki S Chihara Y Ushio M McGarvie LA Burgess AM. The ocular vestibular-evoked myogenic potential to air-conducted sound; probable superior vestibular nerve origin. Clin Neurophysiol. (2011) 122:611–6. 10.1016/j.clinph.2010.07.018 20709596

[17] Murofushi T Matsuzaki M Wu C-H. Short tone burst–evoked myogenic potentials on the sternocleidomastoid muscle: are these potentials also of vestibular origin? Arch Otolaryngol Head Neck Surg. (1999) 125:660–4. 10.1001/archotol.125.6.660 10367923

[18] Jost TA Drewelow G Koziol S Rylander J. A quantitative method for evaluation of 6 degree of freedom virtual reality systems. J Biomech. (2019) 97:109379. 10.1016/j.jbiomech.2019.109379 31679757

[19] Borrego A Latorre J Alcañiz M Llorens R. Comparison of oculus rift and HTC vive: feasibility for virtual reality-based exploration, navigation, exergaming, and rehabilitation. Games Health J. (2018) 7:151–6. 10.1089/g4h.2017.0114 29293369

[20] Spitzley KA Karduna AR. Feasibility of using a fully immersive virtual reality system for kinematic data collection. J Biomech. (2019) 87:172–6. 10.1016/j.jbiomech.2019.02.015 30853091

[21] Faralli M Longari F Ricci G Ibba M Frenguelli A. Influence of extero- and proprioceptive afferents of the plantar surface in determining subjective visual vertical in patients with unilateral vestibular dysfunction. Acta Otorhinolaryngol Ital. (2009) 29:245–50.Available online at: https://www.ncbi.nlm.nih.gov/pmc/articles/PMC2821126/. 20162024 PMC2821126

[22] Nola G Mostardini C Salvi C Ercolani AP Ralli G. Validity of Italian adaptation of the Dizziness Handicap Inventory (DHI) and evaluation of the quality of life in patients with acute dizziness. Acta Otorhinolaryngol Ital. (2010) 30:190. Available online at: https://www.ncbi.nlm.nih.gov/pmc/articles/PMC3008147/. 21253284 PMC3008147

[23] Eljamel S Hussain M Eljamel MS. Should initial surveillance of vestibular schwannoma be abandoned? Skull Base. (2011) 21:59–64. 10.1055/s-0030-1265824 22451801 PMC3312417

[24] Blödow A Helbig R Wichmann N Bloching M Walther LE. The video head impulse test: first clinical experiences. HNO. (2013) 61:327–34. 10.1007/s00106-012-2592-0 23588677

[25] Mahringer A Rambold HA. Caloric test and video-head-impulse: a study of vertigo/dizziness patients in a community hospital. Eur Arch Otorhinolaryngol. (2014) 271:463–72. 10.1007/s00405-013-2376-5 23494283

[26] Perez N Rama-Lopez J. Head-Impulse and Caloric Tests in Patients With Dizziness. Otol Neurotol. (2003) 24:913–7. 10.1097/00129492-200311000-00016 14600474

[27] Jorns-Häderli M Straumann D Palla A. Accuracy of the bedside head impulse test in detecting vestibular hypofunction. J Neurol Neurosurg Psychiatry. (2007) 78:1113–8. 10.1136/jnnp.2006.109512 17220287 PMC2117540

[28] Baloh RW Sills AW Honrubia V. Patients with Peripheral and Central Vestibular Lesions. Ann Otol Rhinol Laryngol. (1977) 86:24–30. 10.1177/00034894770865S303 410351

[29] Black RA Halmagyi GM Thurtell MJ Todd MJ Curthoys IS. The active head-impulse test in unilateral peripheral vestibulopathy. Arch Neurol. (2005) 62:290–3. 10.1001/archneur.62.2.290 15710858

[30] Fernandez C Goldberg JM. Physiology of peripheral neurons innervating semicircular canals of the squirrel monkey. II Response to sinusoidal stimulation and dynamics of peripheral vestibular system. J Neurophysiol. (1971) 34:661–75. 10.1152/jn.1971.34.4.661 5000363

[31] Goldberg JM. Afferent diversity and the organization of central vestibular pathways. Exp Brain Res. (2000) 130:277–97. 10.1007/s002210050033 10706428 PMC3731078

[32] Eza-Nuñez P Fariñas-Alvarez C Perez-Fernandez N. The Caloric Test and the Video Head-Impulse Test in Patients with Vertigo. J Int Adv. (2014) 10:144–9. 10.5152/iao.2014.6434640489

[33] Curthoys IS MacDougall HG Vidal P-P de Waele C. Sustained and Transient Vestibular Systems: A Physiological Basis for Interpreting Vestibular Function. Front Neurol. (2017) 8:117. 10.3389/fneur.2017.00117 28424655 PMC5371610

[34] Ralli M Greco A Altissimi G Turchetta R Longo L D'Aguanno V. Vestibular schwannoma and ipsilateral endolymphatic hydrops: an unusual association. Int Tinnitus J. (2017) 21:128–32. 10.5935/0946-5448.20170024 29336131

[35] Naganawa S Kawai H Sone M Nakashima T Ikeda M. Endolympathic hydrops in patients with vestibular schwannoma: visualization by non-contrast-enhanced 3D FLAIR. Neuroradiology. (2011) 53:1009–15. 10.1007/s00234-010-0834-y 21221556

[36] Chiarovano E de Waele C MacDougall HG Rogers SJ Burgess AM Curthoys IS. Maintaining balance when looking at a virtual reality three-dimensional display of a field of moving dots or at a virtual reality scene. Front Neurol. (2015) 6:164. 10.3389/fneur.2015.00164 26284023 PMC4515556

[37] Parietti-Winkler C Lion A Frère J Perrin PP Beurton R Gauchard GC. Prédiction de la compensation de l'équilibre après la chirurgie du schwannome vestibulaire. Neurorehabil Neural Repair. (2016) 30:395–401. 10.1177/1545968315600270 26253176

[38] Schut IM Engelhart D Pasma JH Aarts RGKM Schouten AC. Compliant support surfaces affect sensory reweighting during balance control. Gait Posture. (2017) 53:241–7. 10.1016/j.gaitpost.2017.02.004 28231556

[39] Nashner LM Black FO Wall C. Adaptation to altered support and visual conditions during stance: patients with vestibular deficits. J Neurosci. (1982) 2:536–44. 10.1523/JNEUROSCI.02-05-00536.1982 6978930 PMC6564270

[40] Creath R Kiemel T Horak F Jeka JJ. Limited control strategies with the loss of vestibular function. Exp Brain Res. (2002) 145:323–33. 10.1007/s00221-002-1110-0 12136382

[41] Vidal P-P Lacquaniti F. Perceptual-motor styles. Exp Brain Res. (2021) 239:1359–80. 10.1007/s00221-021-06049-0 33675378 PMC8144157

[42] Tjernström F Fransson P-A Kahlon B Karlberg M Lindberg S Siesjö P. Different Visual Weighting due to Fast or Slow Vestibular Deafferentation: Before and after Schwannoma Surgery. Neural Plast. (2019) 2019:4826238. 10.1155/2019/4826238 30911290 PMC6398006

[43] Nashner LM Wolfson P. Influence of head position and proprioceptive cues on short latency postural reflexes evoked by galvanic stimulation of the human labyrinth. Brain Res. (1974) 67:255–68. 10.1016/0006-8993(74)90276-5 4470421

[44] Menzies RJ Rogers SJ Phillips AM Chiarovano E de Waele C Verstraten FAJ. An objective measure for the visual fidelity of virtual reality and the risks of falls in a virtual environment. Virtual Real. (2016) 20:173–81. 10.1007/s10055-016-0288-6

[45] Parietti-Winkler C Gauchard GC Simon C Perrin PP. Visual sensorial preference delays balance control compensation after vestibular schwannoma surgery. J Neurol Neurosurg Psychiatry. (2008) 79:1287–94. 10.1136/jnnp.2007.135913 18676407

